# Endovascular stenting of chronic retrohepatic inferior vena cava interruption in Budd-Chiari syndrome

**DOI:** 10.1016/j.jvscit.2026.102296

**Published:** 2026-05-02

**Authors:** Tripti Mathur, Ariyan Tabesh, K. Pallav Kolli, Shahram Aarabi

**Affiliations:** a Department of Surgery, UCSF-East Bay General Surgery Residency Program, Oakland, CA; b Department of Radiology and Biomedical Imaging, University of California San Francisco, San Francisco, CA

**Keywords:** Budd-Chiari syndrome, Chronic BCS, Congenital BCS, Endovascular stenting, Retrohepatic IVC obstruction

## Abstract

Budd-Chiari syndrome is a rare cause of hepatic venous outflow obstruction, with heterogenous etiologies. We report the case of a 35-year-old man with chronic, compensated Budd-Chiari syndrome due to retrohepatic inferior vena cava (IVC) occlusion, likely resulting from early-life thrombosis. Imaging and venography confirmed a 3-cm IVC interruption with extensive alternative venous drainage. Endovascular recanalization, balloon venoplasty, and stent placement successfully restored hepatic venous outflow, leading to improvement in hepatic congestion and complete resolution of symptoms. This case highlights the importance of advanced imaging in diagnosing chronic thrombotic occlusion and demonstrates that preserved hepatic venous drainage from chronic venous rerouting may allow successful IVC recanalization/stenting without the need for transjugular intrahepatic portosystemic shunt placement.

Budd-Chiari syndrome (BCS) is a rare cause of hepatic venous outflow obstruction with diverse etiologies.^1^ Patients present most commonly with abdominal pain, hepatomegaly, and ascites.^2^ Obstruction may occur at any level, ranging from small hepatic veins to the inferior vena cava (IVC), resulting in hepatic congestion, portal hypertension, and liver failure.^3^

In Western countries, classic BCS predominates and is frequently associated with prothrombotic disorders. In contrast, hepatic vena cava BCS is more prevalent in Asian populations, where congenital anomalies and membranous obstruction are observed.^4^^,^^5^ Cases of chronic retrohepatic IVC occlusion causing hepatic vena cava BCS are rarely reported, and descriptions of its endovascular management remain limited. We present a case of chronic, compensated hepatic vena cava BCS in a 35-year-old man caused by retrohepatic IVC occlusion, likely from early-life thrombosis. Written informed consent for publication of this case report and accompanying images was obtained from the patient.

## Case report

A 35-year-old man from Afghanistan presented with an 8-month history of abdominal pain, decreased oral intake, and unintentional weight loss (∼5 kg). His pain worsened with solid food, and he subsisted primarily on juices. In mid-2024, while in Afghanistan, he was noted to have hepatomegaly, diagnosed with BCS, and subsequently underwent paracentesis for ascites in Qatar. After arrival in the United States, further evaluation revealed portal hypertension, with ascites, hepatomegaly, and hematochezia. He was referred to our tertiary center for further treatment and management.

**Fig 1:**
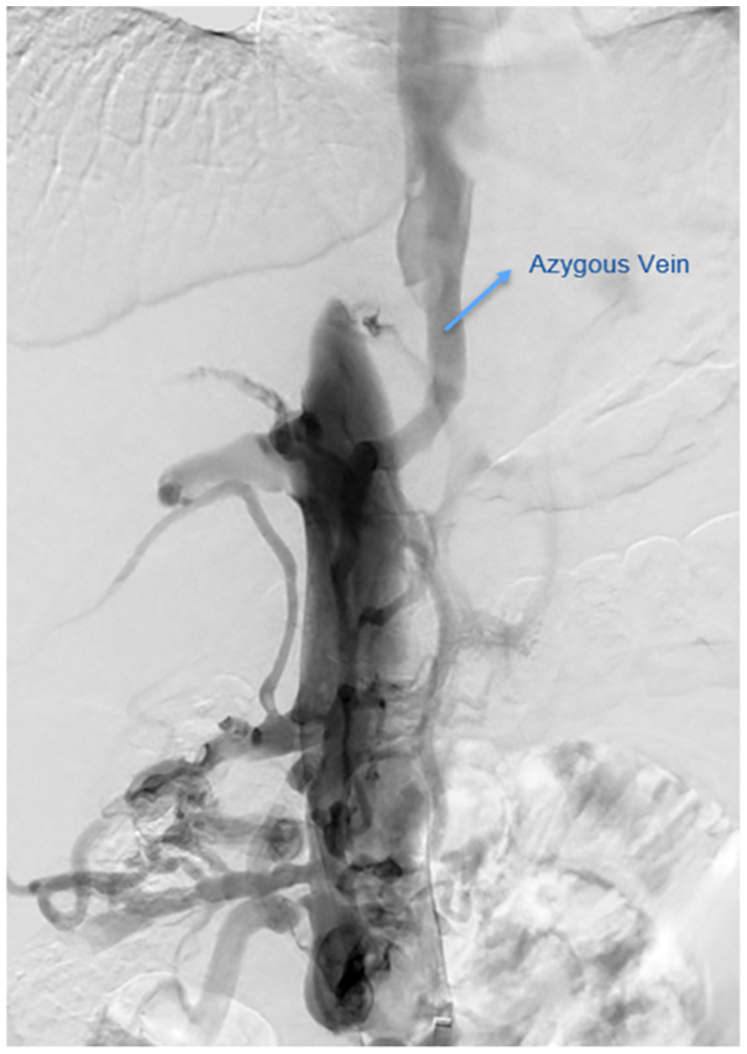
Preintervention venography demonstrating abrupt interruption of the retrohepatic inferior vena cava (IVC) with extensive alternative venous drainage. There is no opacification of the expected intrahepatic IVC segment, and prominent azygos and hemiazygos alternative drainage pathways are visualized, consistent with chronic hepatic venous outflow obstruction due to chronic IVC interruption. Arrow: azygous vein.

Hepatorenal duplex ultrasound examination demonstrated preserved portal venous flow. The right and middle hepatic veins drained normally into the IVC, whereas left hepatic vein drainage was not clearly visualized. The retrohepatic IVC appeared narrowed with absent flow. Cross-sectional imaging findings were consistent with chronic hepatic venous outflow obstruction, including caudate hypertrophy, heterogeneous enhancement, surface nodularity, venous tributaries, ascites, and bowel-wall edema. Venography with intravascular ultrasound (IVUS) examination confirmed a 3-cm interruption of the retrohepatic IVC, with retrograde suprarenal IVC flow and convergence of the bilateral external iliac veins into a large venous channel. Hepatic venous outflow was maintained through tributaries: the azygos and hemiazygos systems provided primary drainage, the right hepatic lobe drained via an accessory hepatic vein entering the IVC at the L2 level, and the left lobe drained through small tributary veins adjacent to the caudate lobe (Fig 1).

Therapeutic anticoagulation with heparin was initiated. After multidisciplinary discussion, the patient underwent endovascular recanalization of the intrahepatic IVC under general anesthesia.

An 8F sheath was inserted via the right common femoral vein. Initial venography demonstrated a short conical configuration of the IVC leading to complete occlusion. A 5F angled catheter and hydrophilic guidewire were advanced to the level of occlusion. Repeat venography revealed small adjacent venous channels and a possible thrombotic cord, suggesting true lumen. The occlusion was crossed bluntly using the angiographic catheter and hydrophilic guidewire, and the right atrial location was confirmed by blood aspiration and venography. Multiple fluoroscopic oblique projections and IVUS examination confirmed the expected IVC course.

**Fig 2:**
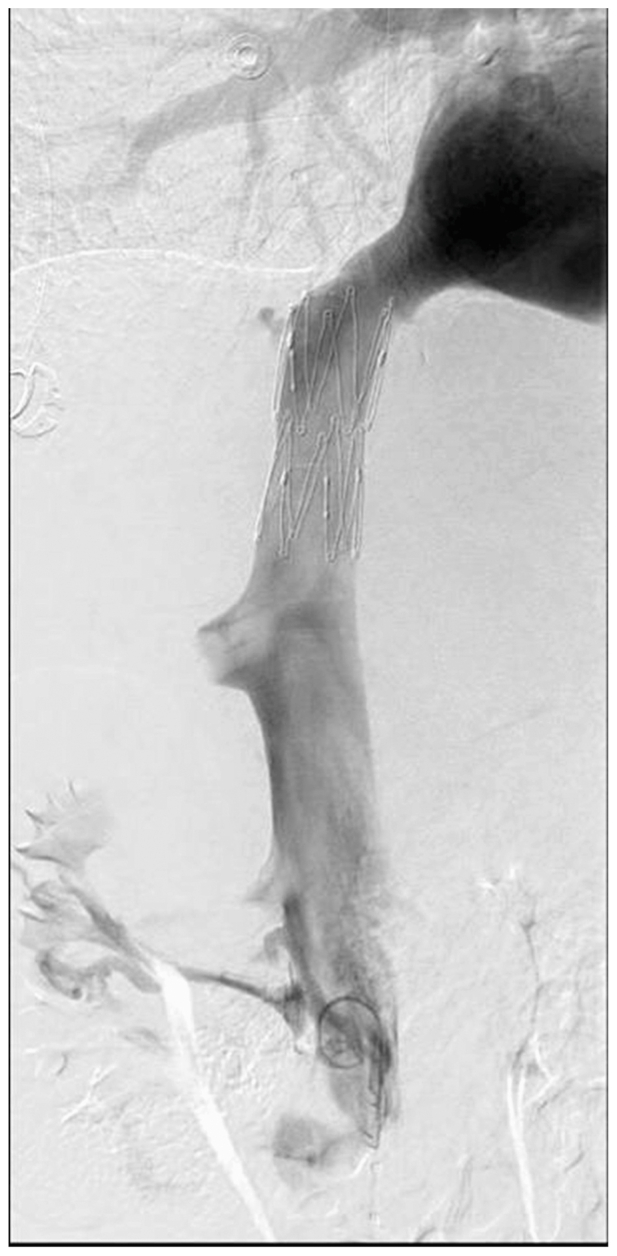
Completion venography following inferior vena cava (IVC) recanalization and stent placement. The image demonstrates successful deployment of a 20 × 50-mm self-expanding bare-metal stent across the retrohepatic IVC occlusion, with excellent stent expansion, brisk antegrade flow, and resolution of collateral venous filling.

Serial venoplasty was performed with 6- and 12- mm Mustang balloons (Boston Scientific Corporation), followed by 16- and 20-mm Conquest balloons (BD/Bard Peripheral Vascular, Inc). Venography after each dilation confirmed successful recanalization without evidence of extravasation. A 20 × 50-mm self-expanding bare-metal Cook Z-stent (Cook Medical) was deployed across the hepatic IVC. This stent was selected for its large interstices to reduce the risk of thrombosis in adjacent tributaries, particularly the large inferior accessory hepatic vein near the caudal implantation site. IVUS examination and venography demonstrated stent expansion, resolution of aberrant tributary liver drainage, and reduction in the trans-IVC pressure gradient from 8 mm Hg to 1 mm Hg. The right accessory hepatic vein remained patent with brisk antegrade flow (Fig 2).

Postoperatively, the patient was transitioned to apixaban 5 mg twice daily on postoperative day 2 and discharged with an initial 6-month plan, later extended indefinitely. At 1-month follow-up, imaging showed patent IVC and hepatic veins, decreased congestion, complete resolution of ascites, and liver stiffness of 8.6 kPa, excluding advanced fibrosis. Thrombophilia workup was negative. Computed tomography venography at 6 months confirmed continued stent patency. The patient remains clinically stable and asymptomatic under surveillance with annual hepatorenal duplex ultrasound examination and computed tomography venography.

## Discussion

BCS represents a rare and heterogeneous spectrum of hepatic venous outflow obstruction with diverse etiologies.^1^ BCS is broadly classified as primary, resulting from intrinsic venous pathology (eg, thrombosis, webs, or endophlebitis), or secondary due to extrinsic compression (eg, tumors, abscess, or cysts).^6^ Primary BCS may be further categorized according to the site of venous obstruction. Classic BCS involves the hepatic veins and typically presents with acute and severe clinical manifestations. In contrast, hepatic vena cava BCS involves the intrahepatic or suprahepatic IVC typically following a more chronic course with comparatively favorable prognosis.^7^

In Western countries, classic BCS predominates and is most commonly associated with underlying prothrombotic states. Conversely, hepatic vena cava BCS occurs more frequently in East and South Asian populations, where congenital IVC anomalies and segmental obstructions are more prevelant.^4^^,^^5^

Chronic BCS may remain clinically silent for years. The patient in this case presented with long-standing abdominal pain, ascites, and weight loss, consistent with compensated chronic BCS. Management of BCS is guided by symptom severity, anatomic considerations, and hepatic function. Medical therapy includes anticoagulation to prevent progression and recurrence,^8^ along with diuretics or paracentesis for ascites. Interventional options include angioplasty with or without stenting for short-segment obstructions, transjugular intrahepatic portosystemic shunt (TIPS) for portal decompression, and surgical shunts or orthotopic liver transplantation in advanced cases.^8^

This case is notable for chronic retrohepatic IVC interruption with extensive alternative venous drainage pathways functioning as physiologic portosystemic shunt. Although the initial appearance suggested congenital interruption, the anatomy was not consistent with classic azygous continuation, which typically involves a single large communication between the IVC and azygous vein rather than numerous small venous channels. The presence of concomitant hepatic vein thrombosis further supported an acquired thrombotic process over classic IVC atresia, although uncertainty remains as to whether this process was truly congenital or was acquired perinatally.

Accurate delineation of the venous anatomy was therefore essential. Venography and IVUS study defined the extent of occlusion, mapped the venous drainage pathways, and demonstrated a residual channel (“string sign”), which allowed successful blunt recanalization and stent placement. In the absence of an identifiable true lumen, sharp recanalization may be required, although this approach is technically more difficult, particularly for longer occlusions.

The presence of extensive alternative venous drainage pattern directly influenced management. Because these pathways partially decompressed the portal system, TIPS was unlikely to provide additional benefit and would have introduced procedural risk. Instead, treatment focused on restoring IVC continuity with recanalization and stenting, thereby preserving native hepatic venous outflow and avoiding permanent shunting. Endovascular stenting restored antegrade hepatic venous drainage, eliminated the transobstruction pressure gradient, and resolved symptoms without requiring TIPS, surgical bypass, or transplantation. Liver elastography further favored the likelihood of long-term hepatic function preservation by confirming the absence of significant fibrosis. Long-term anticoagulation is recommended for all patients with BCS, regardless of whether an underlying prothrombotic condition is identified, to prevent further thrombotic progression.^9^ The patient was transitioned to apixaban, which was well tolerated, and remains free of thrombotic or hemorrhagic complications.

This case highlights the need to maintain suspicion for chronic IVC anomalies in endemic populations; the use of multimodal imaging, including an IVUS scan, to define complex anatomy and guide intervention; recognition of chronic venous adaptations and physiologic portosystemic shunting that may obviate the need for TIPS; and multidisciplinary collaboration to guide individualized endovascular treatment.

## Conclusions

Chronic retrohepatic IVC occlusion is a rare but treatable cause of chronic BCS. Advanced imaging is essential for accurate diagnosis and procedural planning, and endovascular recanalization with stent placement can effectively restore hepatic venous outflow and relieve symptoms, thereby avoiding TIPS or transplant.

## Funding

No funding was provided.

## Disclosures

These authors have no competing interests.
